# The epidemiology of Lassa fever in Ghana: a study on the 2023 Lassa fever outbreak in Ghana

**DOI:** 10.3389/fpubh.2025.1542842

**Published:** 2025-04-28

**Authors:** Kwasi Atweri Akowuah, Magdalene Sarah Ofori, Deborah Pratt, Abigail Abankwa, Evelyn Yayra Bonney, Nancy Enimil, Eric Odei, Theodore Worlanyo Asigbee, Dennis Laryea, Prince Ketorwoley, Juliana Naa Dedei Acquah Amaning, Maame Serwaa Boapea, Stella Bour, Sally-Ann Ohene, Patrick Avevor, John Kofi Odoom, Franklin Asiedu-Bekoe, Patrick Kuma-Aboagye, Francis Chisaka Kasolo, Benjamin Abuaku, Dorothy Yeboah-Manu, Joseph Humphrey Kofi Bonney

**Affiliations:** ^1^Department of Epidemiology, Noguchi Memorial Institute for Medical Research, University of Ghana, Accra, Ghana; ^2^Department of Virology, Noguchi Memorial Institute for Medical Research, University of Ghana, Accra, Ghana; ^3^Korle Bu Teaching Hospital, Public Health Division, Accra, Ghana; ^4^Ghana Health Service, Public Health Division, Accra, Ghana; ^5^World Health Organization (WHO) Country Office, Accra, Ghana; ^6^Department of Bacteriology, Noguchi Memorial Institute for Medical Research, University of Ghana, Accra, Ghana

**Keywords:** Lassa fever, Lassa fever transmission, Lassa fever outbreak, Ghana, West Africa, sub-Saharan Africa

## Abstract

**Background:**

Viral disease outbreak remains a key public health concern given its impact on life and livelihood. Historical data suggests Lassa fever is endemic in several West African countries with sporadic cases occurring elsewhere in the region. In February 2023, Ghana recorded its second outbreak of Lassa fever following that of 2011. The present study sought to document the epidemiology of the recent outbreak in Ghana.

**Methods:**

The study used data from the case investigation forms accompanying samples submitted to Noguchi Memorial Institute for Medical Research between February and March 2023 for Lassa fever testing. Descriptive analysis was used to analyse and report the demographic characteristics. Inferential statistics was used to determine associations between the study variables.

**Results:**

The overall positivity rate was 5.19% out of the 539 samples received. Most cases were confirmed in the Month of February. Positivity rate was higher among females (5.65%). Over 90% of all confirmed cases were from the Greater Accra Region. Case burden for LF was highest among healthcare professionals and also adults aged 25–35 years. The commonly reported symptoms among confirmed cases included headache, intense fatigue, fever, and muscle/joint pain.

**Conclusion:**

Lassa fever was confirmed among 5.19% of the suspected cases. Transmission was largely through contact with the highest burden among healthcare workers which is suggestive of poor infection control procedures. There is a need to build and sustain fundamental public health capacities to recognise, diagnose, report, and respond to risks of public health concern and interest.

## Introduction

In February 2023, Ghana declared an outbreak of Lassa fever. This comes after a decade since Ghana recorded its first cases in 2011 (1–4). Lassa fever is an acute viral haemorrhagic disease caused by the Lassa virus, a member of the family *Arenaviridae* and is endemic in West Africa (5, 6). Other agents from the same family, such as Junin and Machupo viruses, cause similar syndromes, respectively, in Argentina and Bolivia (7).

Human-to-human transmission typically happens through contact with bodily fluids of an infected person and is linked to poor infection control procedures, whereas animal-to-human transmission typically happens after exposure to rodent excreta and secretions such as urine and saliva (8). When symptoms starts to manifest, one becomes contagious and increase in disease severity, which is consistent with the emergence of pharyngeal shedding, vomiting, diarrhoea, and bleeding, as well as rising viral load in bodily fluids (9).

The diagnosis and clinical management of Lassa fever pose a great challenge since early symptoms of the disease are often indistinguishable from that of malaria which is also endemic in West Africa (7). Signs and symptoms typically occur 1–3 weeks after the patient encounters the virus. As reported by Owolabi et al. (7), most infections (approximately 80%), have symptoms that are mild and are often undiagnosed. These include slight fever, general malaise and weakness, and headache. In 20% of infected individuals, however, the disease may progress to more serious symptoms including haemorrhaging (in gums, eyes, or nose, as examples), respiratory distress, repeated vomiting, facial swelling, pain in the chest, back, and abdomen, and shock (7).

The issue of disease outbreaks, particularly those related to viruses, has gained prominence lately, becoming an area of concern in public health (10). Lassa fever has a significant global toll, with imported cases documented in many countries and worries that it could be used as a biological weapon (11, 12).

In Africa, according to Uwishema et al., 300,000 cases of Lassa fever infections are reported annually with an associated death toll of 5,000 deaths (13). Historical data suggests that Lassa Fever is endemic in the West African countries of Nigeria, Sierra Leone, Liberia, Benin and Guinea, with sporadic cases occurring elsewhere in the region. In West Africa, the disease is widespread, affecting an estimated 2 million persons and deaths of 5,000–10,000 persons annually (14). In some other West African countries, it has been reported that 10–16% of people admitted to hospitals in some areas of Sierra Leone and Liberia, every year have Lassa fever, indicating the severe impact of the disease on the population of this region (15).

In January 2023, Nigeria experienced a large outbreak of Lassa fever, with 4,702 suspected cases, five probable cases, and 877 confirmed cases between epidemiological weeks 1 and 15 of 2023 (week ending 16 April). Out of the confirmed cases, 152 deaths representing a case fatality rate (CFR) of 17% were recorded. This was a 20% increase in confirmed cases in comparison with those reported during the same period in 2022 (16). Prior to Nigeria’s outbreak in 2023, Togo reported an outbreak of Lassa fever following the confirmation of a case on 26 February 2022. Their national authorities notified the World Health Organisation (WHO) of an outbreak of Lassa fever after a case, from Takpamba town which borders Ghana, was laboratory-confirmed (16). Ghana, in February 2023, reported an outbreak of Lassa fever. As of the first week of the outbreak in February, the country had recorded a total of 14 confirmed cases with 1 death (1).

According to the WHO’s proposal in the 2005 International Health Regulation (IHR) ratified by United Nations (UN) member states, the threat of infectious diseases requires an effective and efficient transnational coordinated approach for prevention, detection, and response. To recognise, diagnose, report, and respond to risks to public health, nations must build and sustain fundamental public health capacities (17).

Surveillance remains the best approach for the early detection of outbreaks and diagnosis (7). Understanding the factors associated with these infectious disease outbreaks will consequently contribute greatly to rapidly identifying, and promptly responding to these health threats. This study explored the clinical and demographic characteristics as well as documented the scope and epidemiology of Lassa fever cases detected during the 2023 outbreak in Ghana, to enhance the understanding of the disease based on local evidence.

## Materials and methods

The study used data from the case investigation forms accompanying samples submitted to the Noguchi Memorial Institute for Medical Research (NMIMR) between February and March 2023 for Lassa fever testing. These samples were collected from suspected cases meeting the case definition (18) including contacts of confirmed cases. The forms are completed by trained field investigators at various health facilities as part of their sample collection processes. Details captured on the forms included socio-demographic characteristics, clinical signs and symptoms, exposition risk, and laboratory information. The data as provided on the case investigation forms, were entered into a Microsoft Excel spreadsheet.

### Sample processing

Samples transported from the various health facilities were received at the Virology Department in the Advanced Research Laboratories (ARL) of NMIMR for processing. Ribonucleic Acid (RNA) extraction and purification were carried out using the QIAmp RNA kit (Qiagen, Germany) following the manufacturer’s protocol. Polymerase chain reaction (PCR) amplification processes were performed using the Qiagen OneStep RT-PCR protocol targeting the S and L segments of the viral genome with specifically named primers, the S36 and LVS-339-d.

### Statistical analysis

The data was anonymised and analysed to present an epidemiological description of the Lassa fever outbreak in Ghana. Data was analysed using Microsoft Excel Office 16 and STATA version 17. Confirmed cases with incomplete data, in the form of samples without occupation, were labelled and reported as “Unspecified.”

Graphs and tables were generated using Microsoft Excel. The map was generated using Quantum Geographic Information System (QGIS).

Descriptive statistics was used to analyse and report the demographic characteristics in frequencies, and percentages. Inferential statistics was used to determine associations between the study variables using socio-demographic factors as independent or predictive variables, and PCR test results (positive or negative) as the outcome variable.

The age group classification was done according to the Munich Age Classification system (19). Further, the recommendation by the International Journal of Epidemiology was incorporated to subdivide the Adult age group of MACS into mid-decade age grouping (20).

## Results

The total study participants were 539, of which 248 (46%) were females. Majority of the participants (40.18%) were adults aged 24–34 years. The mean age was 32.43 (SD 13.18). Healthcare workers (34.74%) were the majority of the study participants. The highest number of samples were received from the Greater Accra region 353 (65.49%; Table 1).

**Table 1:** Socio-demographic characteristics of suspected Lassa fever cases tested between February and March 2023.

Variable	Frequency	Percentage (%)
Sex		
Male	291	53.99
Female	248	46.01
Age (Months-m and Years-y)		
≤12 m	2	0.44
13 m-2y	9	1.99
3y–5y	11	2.43
6y–11y	6	1.32
12y–17y	15	3.31
18y–24y^*^	55	12.14
25y–34y	182	40.18
35y–44y	105	23.18
45y–54y	37	8.17
55y–64y	25	5.52
≥64y	6	1.32
Occupation		
Artisan	81	28.42
Farmer	9	3.16
Healthcare Worker	99	34.74
Healthcare Support Staff	10	3.51
other civil/public servant	20	7.02
Student	21	7.37
Trade/Business	36	12.63
Veterinary	3	1.05
Retired/Unemployed	5	1.75
Region		
Ashanti	15	2.8
Bono	2	0.37
Central	136	25.42
Eastern	17	3.18
Greater Accra	350	65.42
North East	1	0.19
Oti	1	0.19
Upper East	4	0.75
Volta	5	0.93
Western	4	0.75

* The age group 18–24 was created as a buffer to allow for the incorporation of the mid-decade (10-year) groupings.

The overall positivity rate was 5.19% out of the 539 samples received. Of the 28 confirmed cases, 3 (10.71%) were suspected cases while 25 (89.29%) were contacts of confirmed cases. The positivity rate among females was 5.65% while that of males was 4.81% (Table 2).

**Table 2:** Testing data on suspected Lassa fever cases tested between February and March 2023.

	Total tested	Total positive	Positivity rate (%)	*χ* ^2^	*p*-value
N	**539**	**28**	5.19		
Sex					
Male	291	14	4.81	(1, 539) 0.19	0.66
Female	248	14	5.65		
Age (Months-m and Years-y)					
≤12 m	2	0	0	(10, 453) 4.48	0.92
13 m-2y	9	0	0		
3y–5y	11	1	9.09		
6y–11y	6	0	0		
12y–17y	15	0	0		
18y–24y	55	2	3.64		
25y–34y	182	14	7.69		
35y–44y	105	7	6.67		
45y–54y	37	3	8.11		
55y–64y	25	1	4		
≥64y	6	0	0		
Occupation					
Artisan	81	2	2.47	(8, 284) 19.99	0.01
Farmer	9	0	0		
Healthcare Worker	99	13	13.13		
Healthcare Support Staff	10	3	30		
other civil/public servant	20	1	5		
Student	21	0	0		
Trade/Business	36	2	5.56		
Veterinary	3	1	33.33		
Retired/Unemployed	5	1	20		
Unspecified	255	5	1.96		

The boldened figures represent the total number tested (539) and the total positive number (28).

The highest positivity (9.09%) rate was recorded among children in their early childhood (3–5) years followed by adults aged 45–55 (8.11%) then 7.69% among adults aged 25–34. However, the highest proportion of Lassa fever cases (50%) was recorded among adults aged 25–34.

In terms of occupation, positivity was highest among veterinary (33.33%), and health support staff (30%). Health support staff comprises persons who, although work at a health facility, offer services other than healthcare. This includes cleaners, orderlies, drivers, and the like. Healthcare workers on the other hand recorded a positivity rate of 13.33%. The burden of Lassa fever cases was, however, proportionally higher among healthcare workers (46.43%). The Chi-Square Test of Independence between the various socio-demographic characteristics and Lassa fever test results indicated a statistically significant association between occupation and Lassa fever status with a *p*-value of 0.01 at a significant level of 0.05.

**Figure 1:**
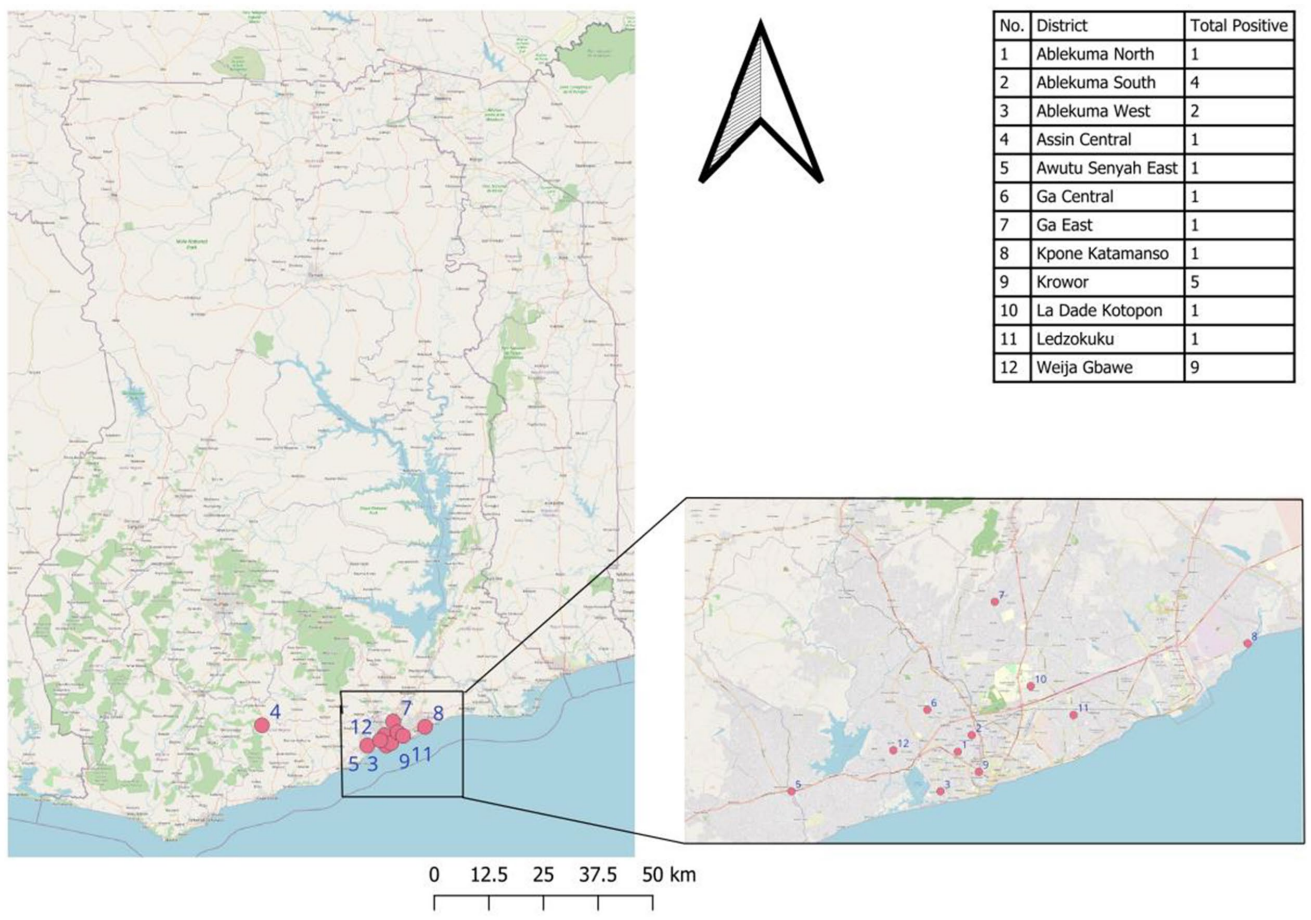
Geographical distribution of Lassa fever cases during the 2023 outbreak in Ghana.

Except for 2 confirmed cases from the central region, all other confirmed cases were recorded in the Greater Accra region (Figure 1).

**Figure 2:**
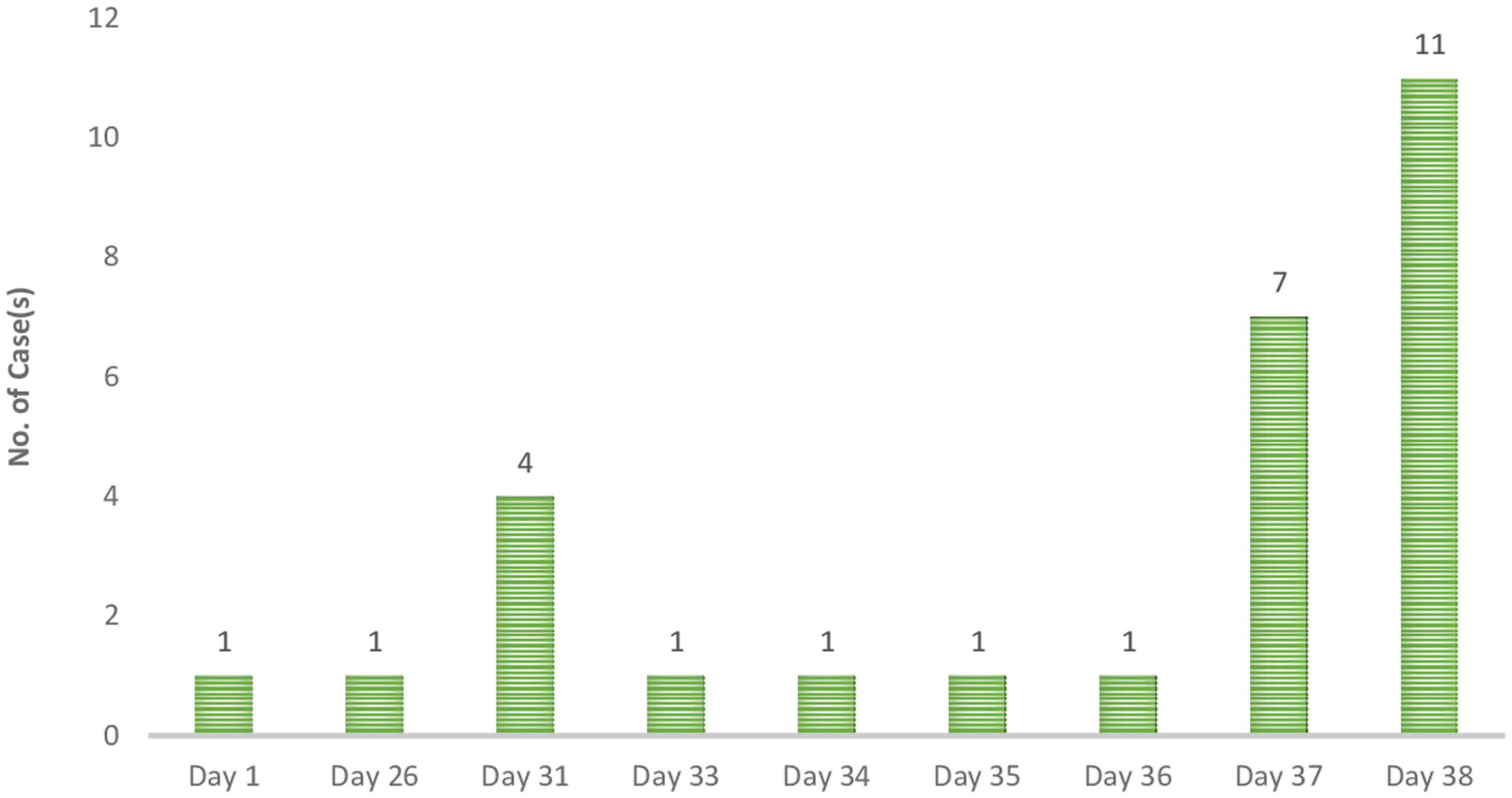
Daily RECORD of Lassa fever case(s) in Ghana (2023).

The highest proportion of confirmed cases was from the Weija-Gbawe district (32.14%) followed by Krowor District (17.85%) and Ablekuma South District (14.29%). A Chi-Square Test of Independence between the reporting region of the suspected case and the Lassa fever test result showed no statistically significant relationship between the two, *χ*^2^ (9, *N* = 538) = 12.47, *p* = 0.19 (Figure 2).

The epi curve portrays an intermittent common source outbreak, which may describe the 2023 outbreak. Using the data from the case investigation form, the actual onset of the outbreak can be said to be 21^st^ January 2023. A surge in cases was recorded on “Day 31” followed by a decline to a single case per day until “Day 37,” when another surge in cases was recorded. The cases peaked on “Day 38” and no case was confirmed subsequently.

The total confirmed cases included 3 suspected cases and 25 contacts. Among the contact cases, the duration between the last contact with a suspected case and the date of sample collection ranged from 1 day to 13 days with the majority (*n* = 14) being 10 days followed by 2 days (*n* = 4).

An analysis of the data on location and timelines of case reporting, either using the date of onset or date seen where applicable, shows that the index case was recorded in the Central region. The patient had a travel history prior to the onset of illness. The next recorded case was on Day 26 in the Greater Accra region. The patient, who was a trader, reported no history of travel or contact with a suspected or confirmed case. All 4 cases recorded on Day 31 were among healthcare workers. All four were reported to have had contact with a case. Two out of the 4 had travelled within the 3 weeks before symptoms onset. The next case was recorded on “Day 33.” This patient, a Banker, was reported to have had contact with a case. The next case was recorded on Day 34 among a health worker who reported a history of contact. This case was followed by two cases on “Day 35” and “Day 36.” The patient from “Day 35” was a health worker. The case on “Day 36” was a Male child in the early childhood years who presented with fever. Additional cases recorded on “Day 37” were mostly health workers or health support staff except for a case reported of a welder who was reported to have been hospitalised but with no record of contact or travel.

The last set of cases (*n* = 11) were all reported on “Day 38.” They all reported no clinical signs or symptoms but had a history of contact. These cases included 4 health workers and 3 health support staff.

### Exposition risk

A Chi-Square Test of Independence was performed to assess the association between exposition risk factors and Lassa fever test results. The exposition risk factors included Hospitalisation (whether the patient was hospitalised or visited anyone in the hospital anytime in the 3 weeks before becoming ill?), Traditional medicine (whether the patient received traditional medicine from a traditional healer?), Funeral ceremonies (whether the patient attended any funeral anytime in the 3 weeks before becoming ill?), Travelled (whether patient travel anytime in the 3 weeks before becoming ill?), Contact (whether patient has had contact with a known suspect case anytime in the 3 weeks before becoming ill?), and Contact with Wild Animal (whether patient had contact with a wild animal, non-human primate or others, that was found dead or sick in the bush, or animal behaving abnormally anytime in the 3 weeks before the illness?).

There was a statistically significant association between traditional medicine use and Lassa fever status as well as Contact with a suspected case at a significant level of 0.05 (Table 3).

**Table 3:** Test of association between exposition factors and Lassa fever.

Risk factor	*χ* ^2^	*p*-value
Hospitalised	(1, 305) 0.55	0.46
Received traditional medication	(1, 294) 4.28	*0.04*
Funeral ceremonies	(1, 254) 0.72	0.40
Travelled	(1, 290) 1.01	0.32
Contact	(1, 299) 10.82	*0.001*
Contact with wild animal	(1, 257) 0.24	0.63

Italicized figure shows significance.

## Discussion

This study set out to document the epidemiology of Lassa fever in Ghana using data from the 2023 outbreak. Out of the total suspected samples of the 539, 28 cases of Lassa fever were confirmed representing an overall positivity rate of 5.19%. This study identified no statistical difference in Lassa fever positivity and biological sex. However, a study by Olayinka et al. (14) reported an increased odds of Lassa fever (LF) among males. This corroborates the observation made when Ghana recorded its first cases of LF, with both being males (4). Yet, LF has been documented to occur among all sexes and ages. Among, the various age groups, positivity rate was highest among the early childhood age group. However, the highest proportion of LF cases was among Adults aged 25–34. Collectively, a proportion of approximately 75% of LF cases were detected among the age group 25–44. Similarly, a 2022 study in Nigeria recorded the highest proportion of LF cases among those aged 25–44.

Based on the case classification, most cases were contacts of confirmed cases. This emphasizes the well-documented means of transmission of LF. This further helps to possibly explain the highest case burden recorded among health workers and health support staff as they may have been exposed in their line of duty. Additionally, this can explain why most of the cases were recorded among young people as the youth are very mobile. From this study, we observed contact with confirmed cases as a factor associated with the transmission of LF. In addition to contact with a confirmed case, there was a significant association between receiving traditional medicine and LF. This could have been a contamination of the packages of the herbal medicine at the place of storage.

Most confirmed cases were from the Greater Accra Region. This can explain the common source intermittent outbreak pattern reflected by the epi-curve. Most of the confirmed cases were from the Weija-Gbawe district which shares a boundary with the central region which could perhaps explain the transmission within the Greater Accra region acknowledging the fact that the index case was a resident of the central region. The cases from the last outbreak of LF in Ghana were recorded in the Ashanti Region.

Uwishema et al. noted the clinical manifestations of LF to include fever and malaise, potentially accompanied by cough, sore throat, headache, nausea, and vomiting (13). Olayinka et al. cited a study by McCormick et al., where they reported a combination of fever, pharyngitis, retrosternal pain, and proteinuria to be significantly associated with LF disease, as similar to the composition of general systemic as well as ear, nose and throat symptoms in their study, which have also been found to be associated with LF disease in Nigeria (13).

The incubation period of LF ranges from 7 to 21 days. Using the duration between the last contact with a case and the date of onset of symptoms for those who developed or date seen at the facility/sample collection as proxy for incubation period, the present study identified a range of 1 day to 13 days which to an extent aligns with that indicated by the WHO according to its 2017 facts sheet on Lassa fever to be between 2 and 21 days (21).

NMIMR was the primary testing facility for LF during the outbreak making the study findings generally representative of the country. However, the data presented is only limited to samples received and tested at the facility during the outbreak period.

## Conclusion

Our data show that the outbreak was concentrated in the Greater-Accra region within the month of February, 2023. The positivity of Lassa fever was found to be higher among veterinary workers. However, in terms of proportion of the positive cases, healthcare workers and health support staff suffered the highest burden. The mode of transmission was predominantly through contact and mostly among healthcare workers and health support staff. The positivity among the veterinary occupation further suggests a potential co-transmission route for zoonoses and human-to-human infections. The findings of the study are suggestive of poor infection control procedures which may be partly due to nonavailability/inadequacy control protocols or failure to take precautionary measures because of perception of risk. The threat of infectious diseases requires an effective and efficient transnational and coordinated approach for prevention, detection, and response. There is therefore the need to build and sustain fundamental public health capacities to recognise, diagnose, report, and respond to risks to public health.

## Data Availability

The original contributions presented in the study are included in the article/supplementary material, further inquiries can be directed to the corresponding author.

## References

[1] Ghana Health service. (2023). Press release: Update on Lassa fever outbreak in Ghana - 28TH February 2023. Available online at: https://ghs.gov.gh/wp-content/uploads/2023/02/lassa-fever-update-1-1.pdf (Accessed: February 25, 2025).

[2] World Health Organization (2022). Marburg virus disease - Ghana. Available online at: https://www.who.int/emergencies/disease-outbreak-news/item/2022-DON402 (Accessed February 26, 2025).

[3] World Health Organization. (2023). Multi-country outbreak of mpox. 1–18. Available online at: https://www.google.com/url?sa=t&source=web&rct=j&url=https://www.who.int/docs/default-source/coronaviruse/situation-reports/20230119_mpox_external-sitrep-14.pdf%3Fsfvrsn%3D365bb443_3%26download%3Dtrue&ved=2ahUKEwjRyoT9uJb_AhUxUqQEHcTpCAwQFnoECB8QAQ&usg=AO (Accessed February 26, 2025).

[4] Dzotsi EK Amankwa J Sarkodie B Thouphique AM Ofei A Oduro J. (2012). The first cases of Lassa fever in Ghana (October 2011). PMC3645162 23661832

[5] Isaac AB Karolina W Temitope AA Joanne E Deborah A Bianca OC. (2022). Prospects of Lassa fever candidate vaccines College of Medicine, University of Ibadan, Ibadan, Nigeria, 2 Polygeia (Global Health student think tank), article history Received: May 11th 2022 revision received: July 2nd 2022 accepted: July 5 th. 2022;16:46–58. 10.21010/Ajid.v16i2S.6 PMC9480887 36124324

[6] Inegbenebor U Okosun J Inegbenebor J. Prevention of Lassa fever in Nigeria. Trans R Soc Trop Med Hyg. (2010) 104:51–4. doi: 10.1016/j.trstmh.2009.07.008 19712954

[7] Owolabi JB. (2016). Re-emerging human viral hemorrhagic fevers: a review re-emerging human viral hemorrhagic fevers: A review (September).

[8] Asogun DA Günther S Akpede GO Ihekweazu C Zumla A. Lassa fever: epidemiology, clinical features, diagnosis, management and prevention. Infect Dis Clin N Am. (2019) 33:933–51. doi: 10.1016/j.idc.2019.08.002, PMID: 31668199

[9] Brosh-Nissimov T. Lassa fever: another threat from West Africa. Disaster Mil Med. (2016) 2:8. doi: 10.1186/s40696-016-0018-3, PMID: 28265442 PMC5330145

[10] Devaux CA. Emerging and re-emerging viruses: a global challenge illustrated by Chikungunya virus outbreaks. World J Virol. (2012) 1:11–22. doi: 10.5501/wjv.v1.i1.11, PMID: 24175207 PMC3782263

[11] Aborode AT Platform HA Ogunsola S. (2020). Demanding of Lassa fever: Reducing its risk as an infectious disease.

[12] Kyei NNA Abilba MM Kwawu FK Agbenohevi PG Bonney JHK Agbemaple TK. Imported Lassa fever: a report of 2 cases in Ghana. BMC Infect Dis. (2015) 15:1–5. doi: 10.1186/s12879-015-0956-2, PMID: 26022703 PMC4448534

[13] Uwishema O Omer MEA Zahabioun A Sablay ALR Tariq R. Lassa fever amidst the COVID - 19 pandemic in Africa: a rising concern, efforts, challenges, and future recommendations. J Med Virol. (2021) 93:6433–6. doi: 10.1002/jmv.27219, PMID: 34289134

[14] Olayinka AT Elimian K Ipadeola O Dan-Nwafor C Gibson J Ochu C. Analysis of sociodemographic and clinical factors associated with Lassa fever disease and mortality in Nigeria. PLoS Glob Public Health. (2022) 2:e0000191. doi: 10.1371/journal.pgph.0000191, PMID: 36962735 PMC10022364

[15] Bausch DG Moses LM Goba A Grant DS Khan H. (2016). Lassa Fever. Viral Hemorrhagic Fevers. 261–286.

[16] World Health Organization. (2022). Lassa fever - Togo. Available online at: https://www.who.int/emergencies/disease-outbreak-news/item/2022-DON362

[17] Keïta M Kizerbo GA Subissi L Traoré FA Doré A Camara MF. Investigation of a cross-border case of Lassa fever in West Africa. BMC Infect Dis. (2019) 19:8–11. doi: 10.1186/s12879-019-4240-8, PMID: 31291900 PMC6621975

[18] WHO. (2022). Lassa fever outbreak toolbox. Available online at: https://cdn.who.int/media/docs/default-source/outbreak-toolkit/final_lassa-fever-outbreak-toolbox_20221011.pdf?sfvrsn=d3852354_1

[19] Althammer A Prückner S Gehring GC Lieftüchter V Trentzsch H Hoffmann F. Systemic review of age brackets in pediatric emergency medicine literature and the development of a universal age classification for pediatric emergency patients - the Munich age classification system (MACS). BMC Emerg Med. (2023) 24:145. doi: 10.1186/s12873-024-01064-0, PMID: 37491219 PMC10369835

[20] Reijneveld SA. Age in epidemiological analysis. Epidemiol Commun Health. (2003) 57:397. doi: 10.1136/jech.57.6.397, PMID: 12775780 PMC1732463

[21] World Health Organisation (WHO). (2017). Lassa Fever.pdf. Available online at: https://www.who.int/news-room/fact-sheets/detail/lassa-fever (Accessed December 5, 2023).

